# Completing the Results of the 2013 Boston Marathon

**DOI:** 10.1371/journal.pone.0093800

**Published:** 2014-04-11

**Authors:** Dorit Hammerling, Matthew Cefalu, Jessi Cisewski, Francesca Dominici, Giovanni Parmigiani, Charles Paulson, Richard L. Smith

**Affiliations:** 1 Statistical and Applied Mathematical Sciences Institute, Research Triangle Park, North Carolina, United States of America; 2 Department of Biostatistics, Harvard School of Public Health, Boston, Massachusetts, United States of America; 3 Department of Statistics, Carnegie Mellon University, Pittsburgh, Pennsylvania, United States of America; 4 Dana Farber Cancer Institute, Boston, Massachusetts, United States of America; 5 Puffinware LLC, State College, Pennsylvania, United States of America; 6 Department of Statistics and Operations Research, University of North Carolina at Chapel Hill, Chapel Hill, North Carolina, United States of America; Indiana University Bloomington, United States of America

## Abstract

The 2013 Boston marathon was disrupted by two bombs placed near the finish line. The bombs resulted in three deaths and several hundred injuries. Of lesser concern, in the immediate aftermath, was the fact that nearly 6,000 runners failed to finish the race. We were approached by the marathon's organizers, the Boston Athletic Association (BAA), and asked to recommend a procedure for projecting finish times for the runners who could not complete the race. With assistance from the BAA, we created a dataset consisting of all the runners in the 2013 race who reached the halfway point but failed to finish, as well as all runners from the 2010 and 2011 Boston marathons. The data consist of split times from each of the 5 km sections of the course, as well as the final 2.2 km (from 40 km to the finish). The statistical objective is to predict the missing split times for the runners who failed to finish in 2013. We set this problem in the context of the matrix completion problem, examples of which include imputing missing data in DNA microarray experiments, and the Netflix prize problem. We propose five prediction methods and create a validation dataset to measure their performance by mean squared error and other measures. The best method used local regression based on a K-nearest-neighbors algorithm (KNN method), though several other methods produced results of similar quality. We show how the results were used to create projected times for the 2013 runners and discuss potential for future application of the same methodology. We present the whole project as an example of reproducible research, in that we are able to make the full data and all the algorithms we have used publicly available, which may facilitate future research extending the methods or proposing completely different approaches.

## Introduction

**Figure 1:**
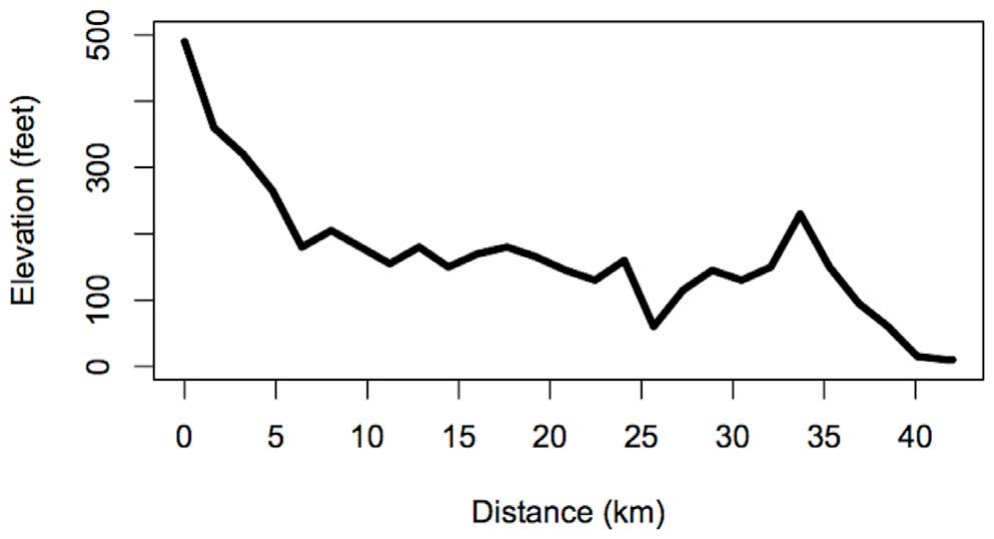
Elevation profile of the Boston Marathon course.

The increasing prevalence of “big data” in all areas of science has led to a focus on statistical prediction algorithms that are appropriate for large systems in many different contexts. Examples include genomics (e.g. trying to decide which genes may be responsible for a disease), high-energy physics (e.g. deciding when irregularities from an experiment such as the Large Hadron Collider may be indicative of a new elementary particle such as the Higgs boson) or climate change (e.g. trying to predict future temperatures, precipitations, hurricane counts, etc., by combining many sources of both observational and climate model data). However, all of these areas require a good deal of scientific expertise to even make sense of the data. Another difficulty is that access to original data sources is often restricted, hindering reproducibility of the resulting research. There has been a trend towards identifying problems for which the data sources and algorithms are freely available, the problem is easily stated in language that does not require advanced scientific expertise, and is sufficiently generic so that a variety of different algorithmic approaches may be applied on the same dataset. The best-known example is the *Netflix prize* dataset [1], which used nearly 100,000,000 ratings by around 480,000 subscribers of nearly 18,000 movies. Despite the large number of ratings, they only represent about 1.2% of the possible subscriber/movie combinations, and the prize competition was essentially to find an algorithm for estimating all the ratings which subscribers would have given to movies they had not seen. The problem discussed in this paper is for a much smaller dataset but has a number of similar features, such as the data are easily made public and the problem is easy to describe, but it is sufficiently complicated allowing for a number of different statistical/algorithmic approaches. The Boston marathon is a running race at the standard marathon distance (42.2 km) which has been run each year since 1897, and which in recent years has had over 20,000 participants; Figure 1 displays the elevation profile of the course. It is the only race, among major marathons, which requires qualifying times of most of its entrants. The race on April 15, 2013, was disrupted by two bombs placed near the finish line, which resulted in three deaths and several hundred injuries. Of lesser concern, in the immediate aftermath, was the fact that nearly 6,000 runners failed to finish the race, the majority of whom would presumably have done so had the race not had to be stopped. It therefore became a priority for the race organizers and the running community to find some way to recognize the achievements of these runners. Shortly after the event, one of the authors (RS) was approached by the Boston Athletic Association (BAA), organizers of the Boston marathon, and invited to propose a procedure for imputing the finish times of all the runners who did not complete the race. The available data consist of “split times” for each of the 5 km sections of the course. The imputation exercise was confined to runners who reached at least the halfway point of the race but did not finish, and for about 80% of those runners, complete split times are available up to 40 km. In other words, the objective is then to predict the runner's split time from 40–42.2 km based on her times for 0–5 km, 5–10 km and so on up to 35–40 km. However, the other 20% of the runners whose times have to be imputed had to drop out at earlier points of the course, and we seek an approach that would also predict those runners' finishing times as accurately as possible. Just as the Netflix prize competition, this may be formulated as an example of what is known as the matrix completion problem [2], [3], which is concerned with finding all the missing entries in a large matrix (for which only a fraction of the entries are available). As an example of statistical approaches to matrix completion [4], proposed a regularized singular value decomposition (SVD) approach. SVD is a standard linear algebra algorithm for representing the entries of a matrix as a linear combination of certain *singular vectors* with weights derived from a sequence of numbers called the *singular values*. The regularization algorithm of [4] implements SVD iteratively with down-weighting of the singular values by soft thresholding (replacing each singular value *d* by 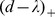 for some constant 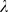) through an algorithm that they call SOFT-IMPUTE. This improves on the SVD-IMPUTE algorithm of [5], which was developed in the context of imputing missing values in a DNA microarray experiment. The latter paper also considered a number of other algorithms, including an algorithm based on finding some number 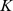 of “nearest neighbors” to the gene for which a prediction is being made, which led to an algorithm which they called KNNimpute. In this paper, we consider some variations of these algorithms, as well as simple linear regression and some others that are more tailored to the specific context of the Boston marathon data, with the intent of comparing their abilities in predicting a validation dataset derived from the results of the Boston marathon in 2010 and 2011. We then use several methods to predict finish times for the runners in the 2013 race and compare the resulting predictions. In addition, because the BAA posted predicted times on their website at http://www.baa.org/races/boston-marathon/participant-information/2013-boston-marathon-news.aspx along with a description of the method they employed, we compare our proposed methods to it. In a separate website http://www.stat.unc.edu/faculty/rs/Bostonwebpage/readme.html, we have deposited all the raw data used in the study, the algorithms used in the analysis, and our predicted finish times for 5,524 of the non-finishing runners from the 2013 race.

## Data

**Figure 2:**
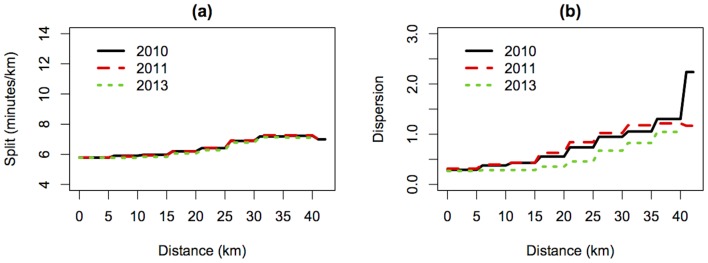
2010, 2011, and 2013 Boston marathon split profile summaries. The point-wise (a) mean and (b) variance for the 2010 and 2011 Boston marathon finishers with finishing times that were slower than 4 hours, and 2013 Boston Marathon racers who made it to the 40 km mark and either (i) did not finish or (ii) had a finishing time slower than 4 hours. These summaries for the marathons are very similar, and the variability increases later in the race.

**Figure 3:**
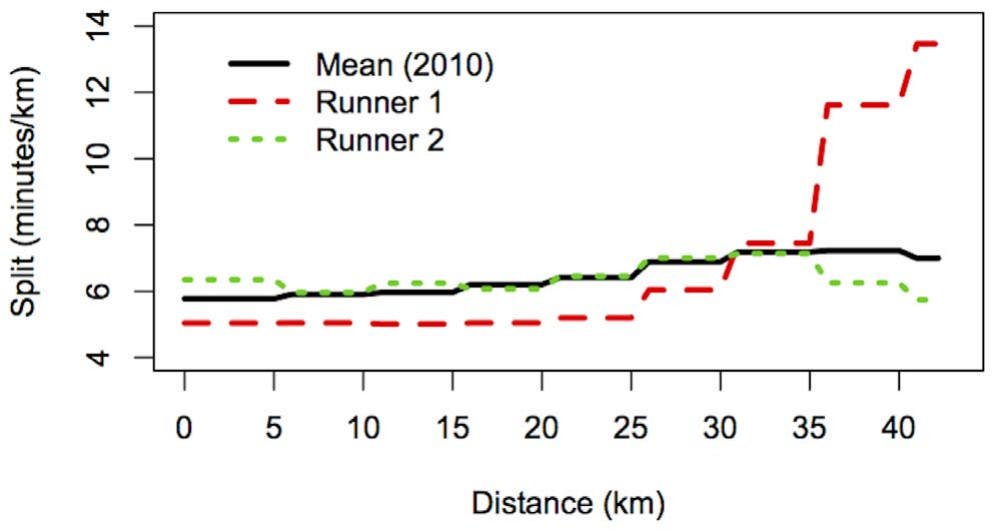
2010 Split profile comparisons. Comparison of split profiles of two runners from the 2010 Boston Marathon along with the 2010 mean from Figure 2.

The data provided to us by the BAA consisted of “split times” for the runners at each of the 5 km intervals on the course (5 km, 10 km, 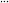, 40 km) as well as finish times for the 17,584 runners who successfully completed the race (three co-authors of this report, FD, DH, and GP are proudly part of this group). In all, there were 5,756 runners who unfortunately did not finish (DNF). In order to provide a comparison dataset for evaluating the quality of our prediction formulas, we asked the BAA for the 2010 and 2011 Boston marathon results (2012 was not considered because that year was unusually hot, and runners slow down much more in hot weather than they do in cool conditions). Therefore, we have the complete results (including split times) for all finishers in those two years, 22,670 in 2010 and 23,913 in 2011. In all, the database for all three years contained 69,923 runners. We did not differentiate runners who may have participated more than once during those three years. The majority of DNF runners were prevented from finishing the race because of the bombs. However, the split times data allowed us to identify some runners who most likely quit the race before the two explosions. The third and last wave of the marathon start was at 10:40am, with the last runner crossing the start line at 10:53am. The two explosions occurred at 2:49pm; based on the latest recorded start time, anyone running faster than 3 h. 56 m. would have finished the race. Therefore we restricted our analysis to runners projected to finish the race in greater than 4 hours, for whom the pattern of split times is typically different from those of runners at the front of the field. More specifically, we excluded runners who finished the race faster than 4 hours in any of the three years and, runners who in 2013 did not finish because they quit before the half-way point (at the 20 km point or earlier). With these reductions, the total dataset for all the three years (2010, 2011, 2013) consisted of 21,930 runners, including 5,628 (25.7%) who were DNFs in 2013. Among those 5,628 DNFs, 93 (1.7%) quit between the 20 km and the 25 km point, 39 (0.7%) between 25 km and 30 km, 459 (8.2%) between 30 km and 35 km, and 533 (9.5%) between 35 km and 40 km. The remaining 4,505 DNF runners (80.0% of all DNFs) passed the 40 km point and therefore had essentially complete splits up to that point. For the few cases in which a runner was not recorded in one split time, presumably due to technical problems, but did show up at a later split, we have interpolated the missing split times. During our initial discussions, members of our group proposed a number of different statistical approaches (described later) for predicting the finishing times, that is, for filling in the missing split times values, including the all-important split from 40 km to the finish. It became clear that we needed an objective method for comparing the quality of predictions of the different approaches. To do this, we created an independent validation dataset, as follows. For the validation dataset, we first excluded the 5,628 DNFs in 2013 from the file of 21,930 runners. Then, we randomly assigned a fraction of 25.7% of the runners in the validation dataset to be DNFs, and set their final time aside so that members of our group would not use it in coming up with their formulas. Moreover, among those runners, we assumed they quit at various points of the course in the same proportion as the true DNFs (1.7% between 20 km and 25 km, 0.7% between 25 km and 30 km, etc.). This therefore created a validation dataset of 17,302 runners that had approximately the same DNF characteristics as the original dataset. We trained the various prediction approaches on the approximately 75% of data with complete time information, and then applied all the proposed statistical approaches to predict the finishing times for the DNF runners in the validation dataset. Lastly, we compared our projected finish times with the true finish times of these runners. In this way, we were able to assess the statistical approaches. Since data from the 2010 and 2011 Boston Marathons will be used to predict the finishing times of the 2013 racers who DNF, it is helpful to see how their results compare to the 2013 Boston Marathon results. Figure 2 displays the point-wise mean and variance for the 2010 and 2011 finishers with times slower than 4 hours and the 2013 runners who made it to the 40 km split and either did not finish or finished with a time slower than 4 hours. We exclude the 2013 runners who did not make it to the 40 km split in order to include more splits in the figure (recall that 80.0% of all DNFs passed the 40 km mark). The mean split profiles for each race are nearly identical, and the corresponding variances are similar as well. The upward trend in the variance plot indicates the increased variability in split times later in the race. The similarity in these figures suggest that use of marathon results from previous years (barring extreme weather conditions in 2012) is reasonable for the prediction of 2013 DNFs. What is not visible in the summary statistics shown in Figure 2, and what makes predicting results for individual runners challenging, is the wide variety of split profiles. For example, there are runners who maintain a steady pace for the entire race, and runners who slow down later in the race. Figure 3 displays the running profiles of two finishers from the 2010 Boston Marathon who illustrate different race patterns. Through split 6 (30 km), the two runners maintain a steady pace, but by the 35 km mark, Runner 1 slows down while Runner 2 speeds up. There are other possible patterns of split profiles, and the desire is for the statistical methodology to capture these different populations of runners and use it for prediction.

## Methods

In this section, we describe the five statistical approaches that were used in our analysis. Some other methods were also explored, but are not included among the main results because we concluded they are not competitive. We also describe the method the BAA selected to use as the projected finishing times and the method proposed by Raymond Britt (http://www.runtri.com/2013/05/unfinished-business-in-boston.html).

### Linear regression

Linear regression is arguably one of the most popular statistical methods (see [6] for a comprehensive introduction). In a broad sense, regression analysis aims to describe the dependence of a quantity of interest on so-called predictor variables. It exists in many variants including linear, nonlinear, simple, multiple, parametric, nonparametric regression among others. The regression variant used in this analysis is multiple linear regression, where multiple refers to the number of predictors and linear to the fact that the regression model is linear in the parameters. The goal is to find the best linear combination of available split times to predict each runner's finish time. In its simplest form, the model used can be written as
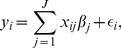
(1)where 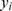 is the sum of the missing split times for runner 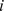, 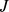 is the number of available split times, 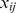 is the available split time for section 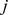 for runner 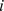, 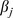 is the coefficient corresponding to split time 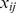, and 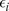 is a random error term assumed to be of mean 0, uncorrelated and with a common variance. We ran regression analyses corresponding to the various possible drop-out points, and then used the analysis to estimate the finish times for the DNF runners in the validation dataset. We also tried a variant of this approach in which age and gender, in addition to split times, were used as predictors of the finish time, which led to only marginal improvements. One interesting finding was that the first two 5 km split times, i.e. the sections covering the first 10 kilometers, had negative coefficients. This implies that on average runners who start out slower have comparatively faster finishing times. To start out slow is advice commonly given to new marathon runners, which seems to be supported by this analysis! A disadvantage of this method is that it doesn't distinguish between different patterns of split times, i.e. the regression coefficients are the same for runners who maintain more or less constant pace across the whole race and for those who slow down a lot during the later stages.

### Nearest neighbor (KNN)

This approach looks at each of the runners who did not complete the race (DNF), and finds a set of comparison runners who finished the race in previous years, whose split times were similar to the DNF runner up to the point where he or she left the race. These runners are called “nearest neighbors.” To turn this idea into more precise estimates we needed to make three basic choices: (1) a method of comparing runners based on split points up to a given stage of the race, (2) deciding how many nearest neighbors to examine, and (3) coming up with a single prediction for the DNF runner based on the different finishing times of the nearest neighbors. For (1), we constructed neighborhoods based on the entire set of splits, by calculating a one-number distance that quantifies how similar the list of splits of the DNF runner is to the splits of a large set of finishers from previous years. For (2) we chose the 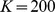 nearest runners – we also tried *K* = 100 and 300, which only changed results slightly and did not make them better (50 did not work well and 500 was computationally inefficient). For (3), the chosen method used a “local linear regression” restricted to the nearest neighbors. In more mathematical detail, the neighborhoods are defined based on the Euclidean distance between the split vectors, and a kd-tree algorithm is used to find the nearest neighbors. As a first step, the kd-tree algorithm places all the runners in the reference database in partitions based on their split times. The partition corresponding to the runner with the missing split times is then identified and the search is limited to that partition and neighboring partitions that could contain runners within the nearest neighborhood. This approach has the advantage that a large percentage of the runners in the database, those with very different split profiles, can be quickly eliminated and the search is conducted over a limited set and hence computationally more efficient than an exhaustive search over all runners in the database. This search algorithm is best suited for applications where the number of database entries is potentially very large, but the dimension of the data is comparatively low, which is the case here. The dimension of the data (the number of split times) is rather small, but the number of runners in the database, especially if more years were included, can be very large. Once the *K* nearest neighbors are found, local linear regression is used to estimate the sum of the missing splits based on the available splits. Local linear regression is chosen to account for cases that have an offset from the profiles in the database. The existence of such cases is somewhat of an artifact as we had limited the database to runners with finishing times over 4 hours. When using a comprehensive database, as would be done for future live marathon time predictions, other methods such as kernel regression or standardizing the nearest neighbor profiles might be preferable based on simplicity and computational speed.

### ANOVA method

The *analysis of variance* or ANOVA method is an adaptation of the well-known statistical technique of the same name [7] to predict runners' finish times in a context where some results are missing. It has been used for a number of years by one of the authors (RS) for determining handicap times in a handicap race, in a context where runners' performances over a number of races are known but with missing data because not every runner runs the same races. In the present context, suppose 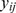 is the logarithm of the split time for runner 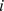 on section 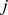 of the course. The simplest two-way ANOVA model represents
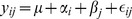
(2)where 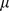 is an overall mean, 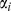 is a parameter due to runner 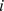, 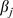 is a parameter due to section 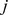, and 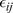 is a random error term (usually assumed to be of mean 0, uncorrelated and with a common variance). A constraint such as 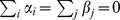 is usually added to make the model (2) identifiable. The reason for taking logarithms is that running times are most naturally modeled multiplicatively: a runner's time on a particular section of the course is the product of one quantity measuring the runner's overall skill and another measuring the length or difficulty of that section. Taking logarithms, and adding a random error, leads to a model of the form (2). The idea is to fit model (2) by the standard method of ordinary least squares, and then apply the resulting estimates of 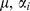 and 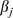 to estimate any missing values. The model (2) assumes homogeneity of the pattern of running times over different sections of the course, which is not appropriate when there are different subgroups of runners with very different profiles. To improve on this, we first break up the runners into different subgroups and apply model (2) separately within each subgroup. The subgroups were defined using two variables: (a) the half-marathon time for each runner, (b) the 20 km to 40 km total time rescaled by dividing by the half-marathon time. The second variable may be thought of as a scaled measure of how much the runner is slowing down. The variable (a) was divided into eight equally sized categories and the variable (b) was divided into four equally sized categories to create 32 subgroups. A number of variants were tried on a precise definitions of the variables (a) and (b) and on the number of subgroups, without substantially affecting the quality of the results.

### SVD approach

Singular Value Decomposition (SVD) finds a set of mutually orthogonal patterns present in a matrix ranked according to strength. These patterns can be linearly combined to approximate missing values in a matrix. It has been used to estimate missing values before, such as in gene expression arrays [5]. Suppose that 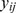 is the split time of runner 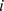 on section 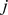 of the course. Using SVD, this runner by section matrix of split times can be factored into
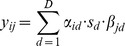
where 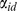 are the left singular vectors, 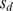 are the singular values, and 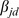 are the right singular vectors. This model separates the effect on the split time of the runners and the course sections. In this model, each row vector 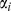 represents runner 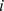, and each row vector 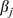 represents course section 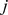. In essence, this model assumes that each split time is the weighted dot product of the runner vector and the course section vector. For example, in the simplest case where 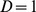, a runner is represented by one number, and a section is also represented by one number. The split time is calculated by multiplying the two numbers and scaling by the first singular value. Faster runners have lower numbers (so the split time is smaller), and harder course sections have larger numbers (so the split time is larger). Once the optimal 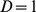 numbers are found, the residuals (the difference between the actual split times and our estimate of the split times) can be calculated. Fitting the residuals in the same manner gives the 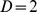 numbers, and so on until any remaining patterns are obscured by noise. A central question in this model is how large 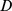 should be. Too small and the accuracy of estimates will suffer, too large and estimates will contain noise. Given the split time data available, different values for 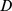 were explored using cross validation, and the value 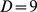 was chosen because it gave the best results. To find missing split times using this model requires three steps. First, the largest possible runner by section matrix is constructed, with the constraint that there are no missing values. This constraint is necessary because SVD requires that matrices have no missing values. The SVD algorithm is run on this matrix, generating estimates of the 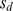 and 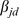 parameters. Second, we estimate each runner vector 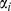 for runners who have missing split times by building a set of linear equations using the known split times for that runner, and the singular values and course section vectors from the first step. These equations are under-constrained, but a minimum norm solution can be found using the Moore-Penrose pseudo-inverse. Finally the estimated runner vectors, together with the estimates from the first step, can be used to calculate the missing split times. The SVD algorithm is central to this method. It is used in the first step to estimate global values for the singular values and the course section vectors. It is used again, implicitly, in the second step to estimate each runner vector since a key step in forming the pseudo-inverse uses the SVD algorithm. This method was implemented in Python 2.7 using the NumPy and Pandas packages. NumPy is used for numerical computing and Pandas is used for data analysis.

### Split-ratio approach

The split-ratio approach is based on estimates of multiplicative constants relating each 5 km segment time to the previous 5 km segment time. We achieve this by taking the mean ratio of each pair of consecutive segment times by gender. These multiplicative constants are then used to predict the remaining time it would have taken a runner to reach the finish line by multiplying the last observed 5 km segment time by the corresponding constant. This proposed method was motivated by the basic extrapolation method proposed by Raymond Britt on http://www.runtri.com/2013/05/unfinished-business-in-boston.html. His rule is very simple: (a) for runners who reached the 40 km split point, multiply their 40 km overall split time by 1.06; (b) for runners who reached the 35 km but not the 40 km split point, multiply their 35 km overall split time by 1.23. Britt did not propose a solution for runners who failed to reach the 35 km point. The split-ratio method is similar to this, except that we use the last observed 5 km segment to predict the remaining running time, whereas Britt uses the last observed split time to predict the full marathon finishing time. By only relying on the last observed 5 km segment, we are able to leverage the most up-to-date information on the runner's last known pace. The multiplicative constants are provided in Table 1. We will now illustrate the differences between an approach that assumes constant pace, the Britt method, and the split-ratio approach. Suppose Mary's last observed split time was 32 minutes for the 30–35 km segment, with an overall split of 3:25:00. She is missing two segment times, the 35–40 km and the 40 km-finish. Under an approach that assumes constant pace, the cumulative race time is multiplied by a constant, 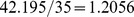, that reflects the assumption that the Mary has and will continue to run a constant pace. Her predicted finish time under a constant pace is 205 times 1.2056 minutes, which translates to 4:07:09. Contrast this with the Britt method, that uses a constant of 1.23, we get a prediction of 4:12:09 for Mary's finishing time. Note how the Britt method accounts for the slowing of the runner through the use of a larger constant, 1.23 versus 1.2056 under a constant pace. Instead of directly predicting the finish time, the split-ratio approach predicts the cumulative time that it would have taken Mary to run these final two segments by multiplying her last observed 5 km split time by the value given in Table 1 (1.4055). This gives us a prediction of 44:58 for her remaining time to finish the course, and an overall time of 4:09:59. In this example, the split-ratio method predicts a finishing time that is between the Britt method and the method that assumes constant pace; however, this will not always be the case. The Britt method will always predict a slower finish than assuming constant pace, but the split-ratio approach can predict a faster or slower finish depending on the pace at the last observed 5 km segment.

**Table 1:** Multiplicative constants used in the Split-ratio approach, estimated from 2010 and 2011 data on finishers over four hours.

	Last 5 km segment completed				
Gender	15 km–20 km	20 km–25 km	25 km–30 km	30 km–35 km	35 km–40 km
Male	5.0648	3.8765	2.5761	1.4333	0.4207
Female	4.8354	3.6965	2.4876	1.4055	0.4230

### Other Methods

In addition to the five main methods described above, we also tried three others which were discarded because they did not perform well in our initial exploratory studies. One method was the SOFT-IMPUTE algorithm of [4], which is implemented in an R package (http://cran.r-project.org/web/packages/softImpute/index.html), and which should in principle be superior to the older SVD approach of [5]. However, it did not perform so well under our cross-validation comparisons. The other methods both had a Bayesian flavor, and consisted of a two-stage regression: (a) for each individual runner, regress split times against either distance or some transformation of distance (polynomial or spline basis functions) to obtain regression coefficients specific to that runner, then (b) the regression coefficients from the first stage are treated as random variables in a second-stage regression which may include additional covariates, such as age and gender. It is not clear why these methods did not perform well, but one possible explanation is that all the standard models for the second stage, part (b) of the model, assume normal distributions of the coefficients, but in this analysis, there are many outliers. It is possible that a different approach, such as nonparametric Bayes in the second stage, could lead to much better results, but in the course of preparing our report for the Boston Marathon, we did not have time to develop this idea.

### Britt method

While investigating the methods discussed above, we also evaluated a basic extrapolation method proposed by Raymond Britt on http://www.runtri.com/2013/05/unfinished-business-in-boston.html. His method is explained above with the Split-ratio approach.

### Constant Pace method

As a final comparison, we also include the approach that was used by the BAA to compute “Projected Finish Time” for each runner when it was eventually posted on the BAA website. This simply took the runner's pace per mile at the last recorded time, and projected that the same pace would continue for the full distance. For example, one runner's final split time was 3:01:32 at the 25 km mark, equivalent to 11 minutes, 42 seconds per mile. Projecting that pace to the full distance (42.195 km or 26.219 miles) leads to a finish time of 5:06:24.

## Results

Each approach introduced above was applied to the DNF runners in the validation dataset. They were then compared with the true finish times of the runners. The following measures were used to compare the approaches:

1. Mean absolute error (mae). We computed the difference between the predicted and actual finish time for each runner, and averaged this difference across all runners. This is the simplest measure of the overall accuracy of a prediction. This average error is reported in minutes.
2. Mean squared error (mse). We computed the absolute difference between the predicted and actual finish time, squared it, and then averaged the squared values. This is similar to computing the variance. This error measure is reported in minutes squared.
3. Proportion of runners for whom the prediction was accurate within 1 minute (1 min).
4. Proportion of runners for whom the prediction was accurate within 2 minutes (2 min).
5. Proportion of runners for whom the prediction was accurate within 3 minutes (3 min).
6. Proportion of runners for whom the prediction was accurate within 4 minutes (4 min).
7. Proportion of runners for whom the prediction was accurate within 5 minutes (5 min).
8. Proportion of runners for whom the prediction was accurate within 10 minutes (10 min).

### Results of the Comparisons in the Validation Data Set

The results of our comparisons of methods are presented in the following sequence of tables. First, we computed each of the above measures of agreement for each of the proposed statistical approaches for the full set of 4,154 DNF runners in the validation dataset. Then, we subdivided the comparisons by gender (M/F), by age (up to or greater than 45), by finish time (up to or greater than 4 hours, 25 minutes), and by the last recorded split (20 km, 25 km, 30 km, 35 km, 40 km). The reasons for the subdivisions by gender, age and finish time was that we had strong prior intuition, which the results confirmed, that the pattern of split times would vary among these subgroups. Tables 2, 3, 4, 5 and 6 display the results, respectively. Note that the Britt method results are only found in the 35 km and 40 km sections of Table 6 because the method only applies to runners who made it to the 35 km split.

**Table 2:** All DNF in validation set.

All(n = 4154)	mae	mse	1 min	2 min	3 min	4 min	5 min	10 min
ANOVA	1.93	18.05	0.528	0.771	0.853	0.896	0.924	0.971
SVD	1.75	14.30	0.577	0.791	0.866	0.901	0.926	0.975
Split-ratio	1.75	14.94	0.584	0.790	0.868	0.903	0.926	0.976
LM	1.64	12.05	0.591	0.804	0.875	0.908	0.931	0.980
KNN	1.57	11.50	0.604	0.801	0.879	0.916	0.941	0.981
Constant Pace	3.25	43.08	0.384	0.625	0.744	0.800	0.840	0.920

**Table 3:** Results by gender.

Gender	Method	mae	mse	1 min	2 min	3 min	4 min	5 min	10 min
F	ANOVA	1.61	10.97	0.581	0.811	0.882	0.918	0.940	0.983
	SVD	1.50	9.93	0.629	0.827	0.888	0.920	0.938	0.982
	Split-ratio	1.46	10.20	0.641	0.830	0.894	0.923	0.940	0.985
	LM	1.39	8.45	0.653	0.841	0.901	0.928	0.947	0.986
	KNN	1.35	7.69	0.651	0.841	0.901	0.928	0.952	0.986
	Constant Pace	2.60	27.26	0.462	0.707	0.796	0.838	0.868	0.939
M	ANOVA	2.31	26.30	0.467	0.725	0.820	0.871	0.905	0.957
	SVD	2.04	19.38	0.517	0.748	0.841	0.880	0.911	0.968
	Split-ratio	2.07	20.47	0.517	0.744	0.838	0.881	0.909	0.966
	LM	1.94	16.25	0.518	0.761	0.846	0.885	0.912	0.972
	KNN	1.82	15.94	0.549	0.754	0.853	0.901	0.929	0.974
	Constant Pace	4.01	61.49	0.293	0.530	0.685	0.756	0.808	0.899

**Table 4:** Results by age.

Age	Method	mae	mse	1 min	2 min	3 min	4 min	5 min	10 min
≤45	ANOVA	1.88	15.84	0.519	0.775	0.860	0.907	0.933	0.974
	SVD	1.73	14.13	0.571	0.794	0.871	0.903	0.927	0.977
	Split-ratio	1.73	15.58	0.592	0.793	0.872	0.905	0.932	0.974
	LM	1.63	11.69	0.589	0.808	0.878	0.911	0.935	0.980
	KNN	1.53	9.55	0.606	0.808	0.882	0.918	0.943	0.982
	Constant Pace	3.02	38.36	0.411	0.663	0.773	0.821	0.853	0.926
>45	ANOVA	1.98	20.27	0.537	0.767	0.846	0.885	0.915	0.968
	SVD	1.76	14.47	0.583	0.787	0.862	0.900	0.924	0.973
	Split-ratio	1.76	14.30	0.576	0.788	0.864	0.902	0.920	0.978
	LM	1.66	12.42	0.592	0.800	0.873	0.905	0.927	0.979
	KNN	1.60	13.46	0.601	0.793	0.876	0.914	0.939	0.979
	Constant Pace	3.48	47.80	0.357	0.588	0.716	0.779	0.827	0.914

**Table 5:** Results by finishing time.

Finish Time (mins)	Method	mae	mse	1 min	2 min	3 min	4 min	5 min	10 min
≤265	ANOVA	1.55	8.42	0.570	0.804	0.881	0.918	0.942	0.983
	SVD	1.49	8.56	0.615	0.810	0.882	0.914	0.936	0.984
	Split-ratio	1.45	7.77	0.619	0.814	0.891	0.923	0.944	0.984
	LM	1.43	7.06	0.622	0.820	0.887	0.914	0.939	0.988
	KNN	1.29	5.18	0.645	0.823	0.895	0.928	0.953	0.990
	Constant Pace	2.80	28.03	0.417	0.653	0.769	0.821	0.856	0.937
>265	ANOVA	2.39	29.51	0.478	0.732	0.820	0.870	0.903	0.957
	SVD	2.06	21.12	0.532	0.768	0.847	0.886	0.914	0.964
	Split-ratio	2.10	23.47	0.542	0.762	0.840	0.879	0.905	0.967
	LM	1.89	17.99	0.554	0.784	0.861	0.900	0.921	0.969
	KNN	1.90	19.02	0.555	0.774	0.860	0.901	0.926	0.969
	Constant Pace	3.78	60.98	0.345	0.593	0.715	0.775	0.821	0.901

**Table 6:** Results by last recorded split.

Last Split	Method	mae	mse	1 min	2 min	3 min	4 min	5 min	10 min
20 km	ANOVA	14.66	401.10	0.016	0.129	0.161	0.226	0.274	0.435
	SVD	9.74	161.35	0.048	0.097	0.194	0.258	0.290	0.613
	Split-ratio	8.41	144.24	0.065	0.097	0.258	0.403	0.435	0.742
	LM	7.89	124.41	0.065	0.258	0.339	0.387	0.435	0.790
	KNN	8.95	198.78	0.081	0.161	0.242	0.290	0.355	0.758
	Constant Pace	20.83	652.38	0.000	0.000	0.032	0.048	0.097	0.226
25 km	ANOVA	9.90	173.13	0.069	0.172	0.172	0.207	0.310	0.655
	SVD	8.33	121.20	0.103	0.138	0.276	0.276	0.379	0.655
	Split-ratio	7.41	107.66	0.034	0.138	0.310	0.517	0.552	0.793
	LM	6.84	97.14	0.172	0.276	0.310	0.310	0.414	0.862
	KNN	7.60	108.27	0.138	0.172	0.310	0.414	0.483	0.724
	Constant Pace	18.54	473.51	0.000	0.000	0.000	0.034	0.069	0.172
30 km	ANOVA	5.60	78.76	0.143	0.296	0.411	0.538	0.631	0.857
	SVD	5.77	82.22	0.131	0.255	0.395	0.513	0.599	0.866
	Split-ratio	5.60	81.32	0.162	0.309	0.436	0.529	0.627	0.860
	LM	5.37	66.98	0.140	0.258	0.401	0.519	0.608	0.873
	KNN	4.58	54.11	0.191	0.331	0.494	0.599	0.707	0.901
	Constant Pace	12.20	248.16	0.035	0.076	0.140	0.172	0.226	0.490
35 km	ANOVA	3.40	25.47	0.244	0.451	0.602	0.713	0.809	0.954
	SVD	3.27	25.15	0.262	0.453	0.634	0.747	0.830	0.954
	Split-ratio	3.26	28.70	0.306	0.494	0.660	0.747	0.809	0.945
	LM	3.11	22.85	0.287	0.501	0.657	0.754	0.834	0.949
	KNN	2.76	17.60	0.294	0.529	0.687	0.802	0.874	0.959
	Britt	4.19	34.89	0.189	0.347	0.497	0.609	0.710	0.920
	Constant Pace	6.46	69.59	0.099	0.172	0.264	0.366	0.467	0.811
40 km	ANOVA	1.08	2.80	0.616	0.875	0.947	0.973	0.985	0.997
	SVD	0.96	2.75	0.675	0.904	0.959	0.976	0.986	0.998
	Split-ratio	1.01	3.61	0.675	0.893	0.952	0.972	0.982	0.997
	LM	0.94	2.59	0.687	0.910	0.964	0.980	0.988	0.998
	KNN	0.94	2.32	0.697	0.899	0.957	0.977	0.987	0.998
	Britt	1.28	3.55	0.524	0.825	0.929	0.969	0.981	0.996
	Constant Pace	1.52	5.00	0.465	0.754	0.884	0.937	0.968	0.995

**Figure 4:**
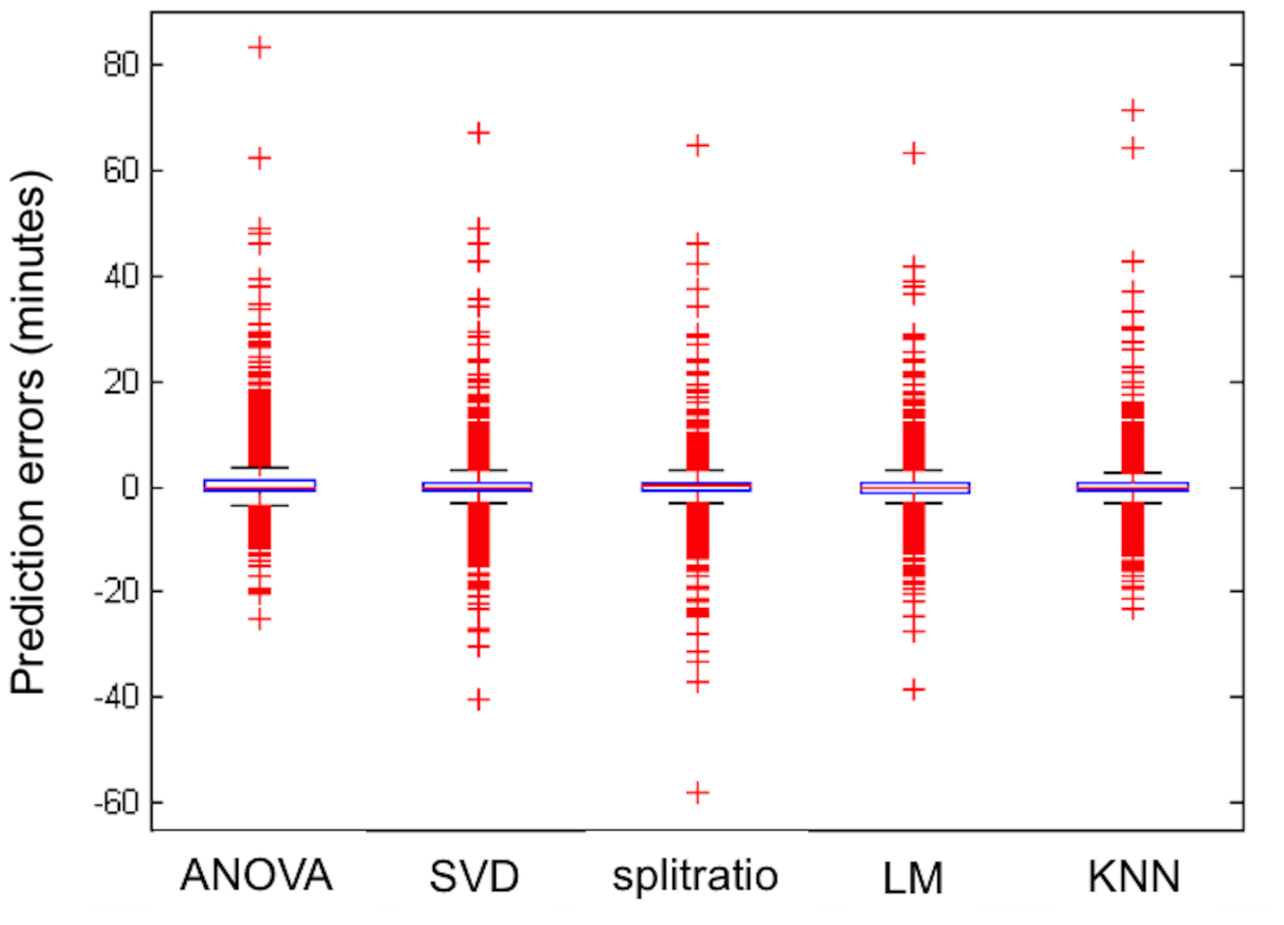
Prediction error box plots. Boxplots of prediction errors in the validation dataset: the ‘box’ in the middle includes 50% of runners. Red marks are individual very large errors.

**Figure 5:**
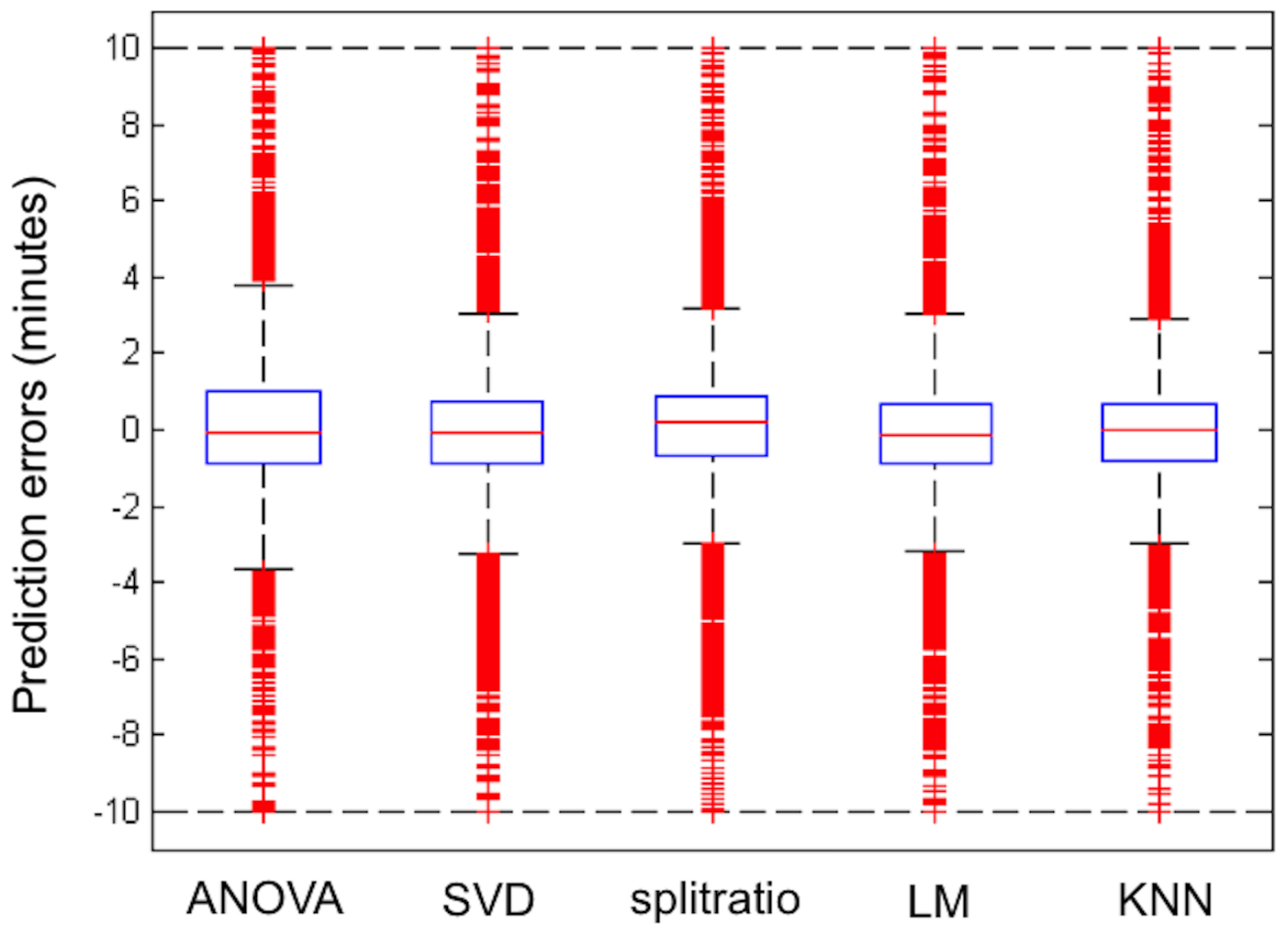
Restriction prediction error box plots. Boxplots of prediction errors in the validation dataset, restricted to runners for whom the prediction error was less than 10

The prediction errors in the validation dataset for each of the five main methods are also displayed in Figure 4. It emphasizes the challenges of predicting the time of these over-four-hour runners. While the majority of errors are tightly packed around zero, there remain runners who slow down, or pick the pace back up in ways that are challenging to infer based on their prior pattern of splits. For the nearest neighbor approach, there remain examples of runners who were one hour slower than we predicted, and also 25 or so minutes faster. Most of those correspond to runners who had to drop out early in the race and are therefore very hard to predict accurately. As can be seen from the tables of results, around 98% of all runners are predicted with an error of less than 10 minutes. To more clearly compare methods, we redrew the box plots in Figure 4 to show only the 98% or so of runners with an error of less than 10 minutes (see Figure 5). Judging by the width of the central box, the ANOVA method has the widest variability (and thus the largest errors) among these five but the other four are hard to distinguish (as noted earlier, we also considered and rejected some methods that had substantially larger prediction errors).

### Analysis of the predictions for 2013

**Figure 6:**
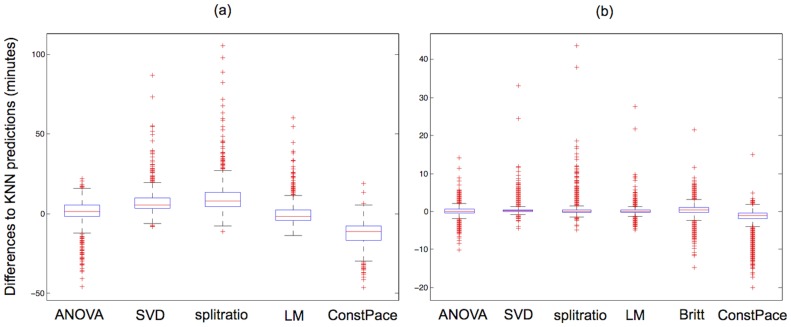
2013 prediction error box plots. Boxplots of differences in predicted finishing times between the KNN method and other methods for participants in the 2013 Boston marathon, who passed the half-marathon mark, but did not complete the course. Predictions are based on (a) splits available up to 30 km or less (n = 515) and (b) splits available up to 35 km or 40 km (n = 5009). Note the scale difference between the two plots.

From the individual runner's perspective, an interesting question is whether using different prediction methods makes a practical difference in the predicted finishing times. By comparing the predicted finishing times for the 2013 Boston marathon, the answer is that for many runners it does not matter which prediction method is used – for over 80% of the runners the differences among methods are less than 5 minutes and only 5% of the runners have differences larger than 20 minutes. Adhering to the mantra of the passionate runner that every minute counts, however, it is worth investigating under which circumstances the methods differ and if these differences are systematic. Figure 6 shows the differences between the KNN predictions and those from the other methods grouped by available split times for the 2013 race. As expected, the differences are larger for predictions based on fewer splits: the width of the blue boxes encompassing the middle 50% of the differences are wider when fewer splits are available, and the overall range of the differences are considerably larger. A feature visible in both plots is that the predicted times using the Constant Pace method are overall systematically lower than the predictions for the other methods. This is not surprising since the Constant Pace method makes no allowance for the fact that most runners slow down in the later stages of the race, though it should be pointed out that especially after the 35 km point (after Heartbreak Hill) some runners do speed up and this explains why the projections of the Constant Pace method are not uniformly faster than the others. Considering the Constant Pace and Britt methods, Figure 6 suggests the variability in predictions are similar, but the Constant Pace method appears to have a downward bias (i.e. the Constant Pace method generally predicts faster finishing times). Given this observation, if one were to resort to a comparatively simple method, using a method along the lines of the Britt method would be the better route rather than using the assumption of constant pace. Even though the Britt method doesn't account for an individual runner's split profile and only uses a simple multiplication factor, that factor is slightly higher than if it were only based on the distance (i.e. the Britt multiplicative factor incorporates the Boston course profile and the slow down of most runners over the later stages of a marathon better than a Constant Pace assumption). On the other hand, the Split-ratio method is only based on the latest available split time. The consequence of using only the latest split is that it can lead to sporadic predictions for runners who significantly change their pace. In Figure 6, the Split-ratio method has more extreme predictions in the high-end indicating that the predictions are higher than those from the other methods (i.e. the Split-ratio method generally predicts slower finishing times). This lack of symmetry is intuitively consistent with the fact that only few runners speed up in the later stages of a marathon, and then only by a comparatively small amount, while more runners slow down (some of them by a significant amount). Hence a method that is solely based on the last split can result in some very high predicted finishing times even though it performs well for the typical runner. To try to understand better the relations between the different methods, we also computed correlations among the projected times, as follows:

1. For each of *j* = 4, 5, 6, 7, 8:
  - For each runner whose last recorded split was at the 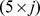-km point, estimate the projected time from that point until the end of the race by each of the prediction methods,
  - Compute the correlation matrix, call it 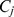, among those projected times.
2. Compute the overall correlation matrix 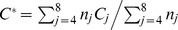, where 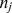 is the number of runners whose last recorded split was at the 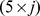-km point.
3. For correlations between the Britt method and any of the others, the same calculation is made but restricted to 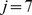 and 8.

This calculation produces the correlation matrix shown in Table 7. Based on this table, we conclude:

**Table 7:** Correlations between predictions of different methods.

	ANOVA	SVD	Split-ratio	LM	KNN	Constant Pace	Britt
ANOVA	1.00	0.84	0.80	0.83	0.84	0.77	0.77
SVD	0.84	1.00	0.98	0.97	0.94	0.75	0.75
Split-ratio	0.80	0.98	1.00	0.97	0.93	0.63	0.63
LM	0.83	0.97	0.97	1.00	0.92	0.68	0.69
KNN	0.84	0.94	0.93	0.92	1.00	0.69	0.68
Constant Pace	0.77	0.75	0.63	0.68	0.69	1.00	1.00
Britt	0.77	0.75	0.63	0.69	0.68	1.00	1.00

1. The correlation between the Britt method and Constant Pace is 1 (which it should be, because for each of 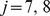, one method is a constant multiple of the other).
2. All four of the SVD, Split-ratio, LM and KNN methods are very highly correlated with each other (correlation above.92).
3. The correlations between the ANOVA method and any of the others, or between any of the SVD, Split-ratio, LM and KNN methods and either of the Britt or Constant Pace methods, are substantially lower (though all the correlations are still positive – above 0.6 – and highly statistically significant).

**Figure 7:**
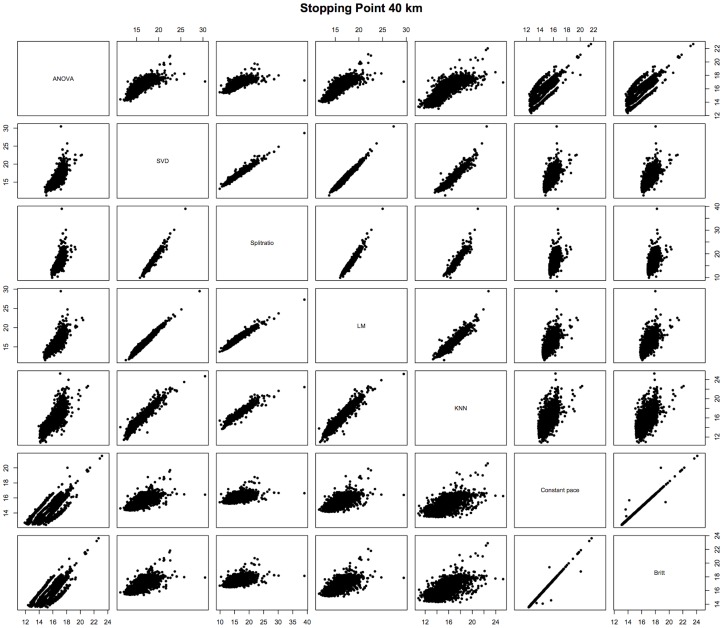
2013 Prediction comparisons. Plots of the predicted 40's predicted 40 km to finish times on the horizontal axis and the SVD method's predicted 40 km to finish times on the vertical axis.

Thus, it appears that the prediction methods are essentially in three groups, one consisting of the ANOVA method, a second consisting of the SVD, Split-ratio, LM and KNN methods, and a third consisting of the Britt and Constant Pace methods. Since the second group of methods appears best under the various statistical measures we have used to evaluate them, this reinforces that any of the four methods may be used roughly interchangeably. In particular, since this group includes the Split-ratio method, which uses *only* the last recorded split time before the runner stopped, this suggests that most of the information useful for prediction is contained in that last split time. Figure 7 provides a way to visualize the connections between the seven methods. The subplots of the figure display the predicted times for the corresponding methods listed along the diagonal of the scatterplot matrix. For example, the scatterplot in row 2 and column 1 has the ANOVA method's predicted 40 km to finish times on the horizontal axis and the SVD method's predicted 40 km to finish times on the vertical axis. Methods that had similar prediction times will have points in the plot very close to a line (e.g. the plot in row 7 and column 6 displaying the predicted times for the constant Pace and Britt methods); plots with more scatter along a line suggest more variability between the methods (e.g. the plot in row 6 and column 7 displaying the predicted times for the KNN and constant Pace methods). Two methods would have identical predictions if the points were exactly on the 45 degree line. As indicated above, these plot suggest three groupings of methods: (i) the ANOVA method, (ii) SVD, Split-ratio, LM, and KNN methods, and (iii) Constant Pace and Britt methods. The ANOVA method stands alone when looking across row 1 and down column 1 - there is a lot of scatter along a line suggesting that the ANOVA method's prediction are not similar to the others. The interior four-plot by four-plot square comparing the SVD, Split-ratio, LM, and KNN methods only have moderate amounts of scatter, and the bottom two-by-two square of the Constant Pace and Britt methods have very little scatter. It is intriguing that there are 22 runners for whom their predicted finishing times by the various methods differed by more than one hour, which appears to be a large difference. A closer investigation of these cases revealed one common characteristic: they all slowed down their pace by at least 3 min/mile. Slowing down is not part of any standard marathon race strategy, but rather an ominous sign. While there are no “true” finishing times, since these runners did not continue, to evaluate the different predictions, it is questionable whether such an evaluation would be meaningful given that all the methods presented here are geared towards more typical profiles and not particularly suitable for these extreme scenarios. It might make sense to exclude such cases altogether.

## Looking Forwards: A Method for Predicting Marathon Finish Times from Intermediate Split Times

Looking beyond the specific issues posed by the 2013 Boston marathon, in this section we discuss the broader implications of a method for predicting finishing times in marathons and other road races from split times taken at intermediate points along the course. Many large road races, including the Boston marathon, provide real-time information on competitors' performances. While the race is in progress, one can go to the race website and look up the split times of any competitor, and it is also possible for friends and relatives to sign up to receive updates by text messaging or email during the race itself. This information is valuable to spectators following the race and to friends or relatives planning to meet their runners at the finish line. Such updates often include estimates of a runner's finish time, but in all cases that we are familiar with, the projection is based on “constant pace” — in other words, the assumption that a runner's pace at the intermediate split time will remain constant for the remainder of the race, which is unrealistic for the majority of runners. The methodology in this paper could, we believe, provide for more realistic estimates, including some indication of uncertainty. For this section, we propose a modified and somewhat simplified version of the “KNN” methodology, which we have found to be as good or better than all the other methods considered. This is a “rescaled KNN” analysis, which works as follows:

1. For a given runner observed at an intermediate point of the course, find 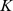 “nearest neighbors” among the database of runners (e.g. who have completed the course in previous years) for whom complete split times are available. As in the earlier analysis, the definition of nearest neighbors is based on Euclidean distance applied to the complete vector of split times up to the sampling point.
2. For each of the nearest neighbors, *rescale* the split times (by multiplying all the split times by a constant) so that the neighbor's cumulative time at the sampling point equals that of the runner being predicted.
3. Project all the split times of the neighbor runners forwards to the finish of the race.
4. The median finish time of all the neighbor runners may be taken as a point prediction of the finish time of the runner being predicted. The distribution of finish times of the neighbor runners may also be taken as a measure of uncertainty — for example, the 5th and 95th percentiles of the neighbor runners may be taken as the lower and upper bounds of a 90% prediction interval for the runner being predicted.

**Figure 8:**
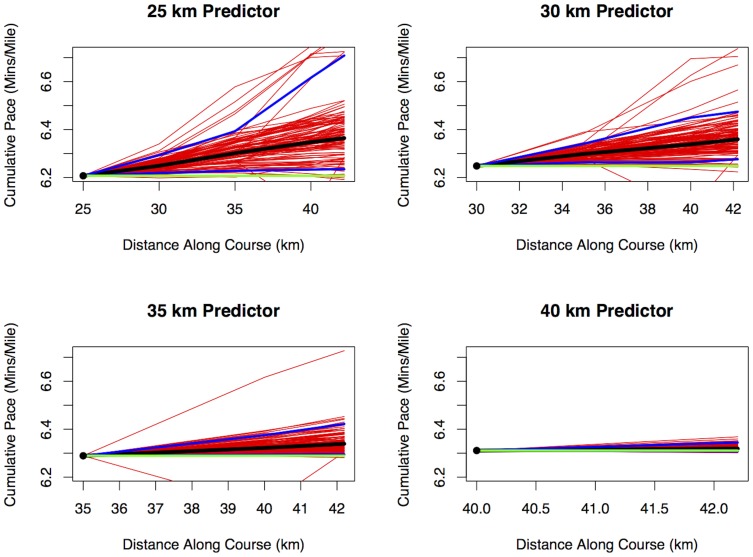
Illustration of Rescaled KNN Method. The figure shows how the method is to predict the pace during the remainder of the race for a runner sampled at the 25*K* = 100 nearest neighbors among the full population of runners, using all the split times up to the sampling point. These neighbors are rescaled by multiplying/dividing by a constant so that the net pace at the sampling time is the same as the runner being predicted. The 100 runner profiles are then shown for the remainder of the course. The think black line is the median through all the neighbor runners and therefore provides a prediction for the runner of interest. The lower and upper blue lines represent the 5th and 95th percentiles of the distribution — these therefore provide the bounds of a 90% prediction interval for the future pace of the runner of interest. The horizontal green line is the Constant Pace projection. All results are presented as net overall pace (minutes/mile) to facilitate comparisons over the length of the course.

**Figure 9:**
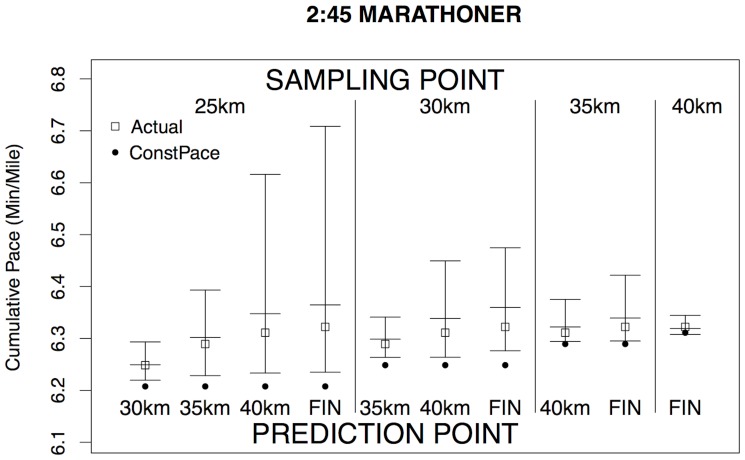
Comparison of Predicted and Actual Pace I. The comparison is made for the 2:45 marathoner depicted in Figure 8. Given the runner's splits up to a sampling point (one of 25 km, 30 km, 35 km or 40 km), the diagram shows the median prediction (central horizontal line) and the boundary points of a 90% prediction interval (outer horizontal lines) for all subsequent split points. Also shown are the runner's actual results (open square dots) and the projections assuming Constant Pace (round black dots). All results are presented as net overall pace (minutes/mile), as in Fig. 8.

**Figure 10:**
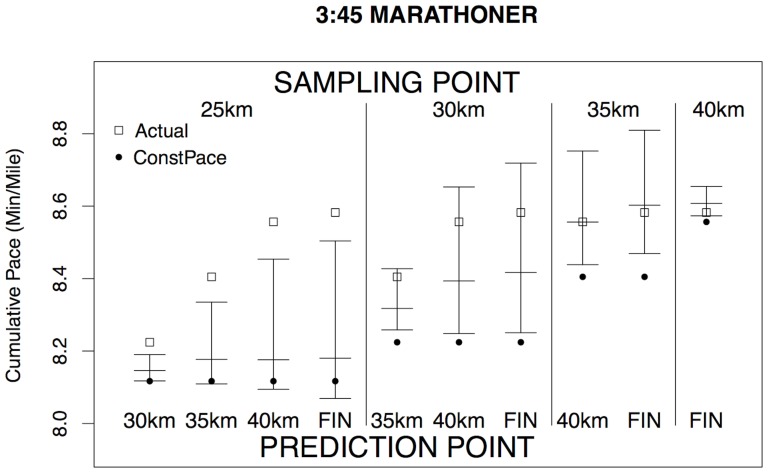
Comparison of Predicted and Actual Pace II. Similar to Fig. 9 but for a 3:45 marathoner.

Figure 8 illustrates this method graphically. This method differs from the KNN method presented earlier in the paper because the rescaling proposed at Step 2 is simpler than the local linear regression step used earlier. Table 8 shows three measures of performance for each of the possible dropout points and five different values of 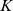. Table 8 also incidentally illustrates the possibility of using the validation sample to choose the optimal 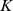. Which 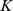 is best among the five values considered varies according to the dropout point and which of the three validation measures is used (MAE, MSE or CovPr), but overall either 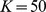 or 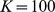 seems fully satisfactory in comparison to the others. Figure 9 illustrates the performance of this method for one runner, whom we have taken to be a runner from the 2013 Boston marathon with a 2:45 finish time, which is well under the Boston qualifying time for his age group (currently 3:05 for a male runner aged 18 to 34), but not an elite runner. This individual ran a fairly consistent pace but slowed slightly in the later stages, which is common among experienced but not elite marathon runners. For this runner, his actual splits (shown by square dots) all fall well within the 90% prediction interval for each of the intermediate times, but the Constant Pace projections fall outside those prediction intervals. This reinforces our argument against using Constant Pace projections, though we have noted earlier that almost all current projections are based on this assumption. Figure 10 illustrates the contrasting performance of a runner who finished in 3:45 with a substantial slowing down in the later stages of the race. In this case, the prediction intervals from a 25 km intermediate sampling point fail to include this runner's actual performances during the later stages of the race, as must inevitably happen for some runners whose pattern of split times differs substantially from the norm. However, the later projections do incorporate this runner's substantial slowing down between 25 km and 35 km, as the prediction intervals from a sampling point after 30 km include the actual runner's performances. In conclusion, the rescaled KNN method seems to be a simple method to define and to implement. This could be recommended as an all-purpose approach to the problem of making real-time projections of finish times in road races, which includes uncertainty bounds and is clearly superior to the simple Constant Pace approach.

**Table 8:** Properties of the rescaled KNN procedure.

Dropout point	Number of dropouts	*K*	MAE	MSE	Coverage Probability
20 km	62	25	9.03	175.3	0.84
		50	8.80	172.7	0.90
		100	8.79	170.8	0.90
		150	8.81	169.6	0.89
		200	8.84	170.5	0.90
25 km	29	25	7.55	105.2	0.76
		50	7.27	103.3	0.83
		100	7.38	105.0	0.86
		150	7.29	104.8	0.90
		200	7.20	101.7	0.86
30 km	314	25	4.76	60.8	0.86
		50	4.75	62.8	0.88
		100	4.83	63.6	0.89
		150	4.87	65.2	0.89
		200	4.91	66.6	0.89
35 km	435	25	2.87	20.2	0.82
		50	2.86	19.6	0.85
		100	2.87	19.5	0.87
		150	2.88	19.3	0.88
		200	2.90	19.6	0.88
40 km	3314	25	0.95	2.31	0.82
		50	0.95	2.31	0.85
		100	0.95	2.29	0.86
		150	0.96	2.32	0.87
		200	0.96	2.34	0.88

For five values of *K*, the table shows the mean absolute error (MAE), mean squared error (MSE) and coverage probability of the proposed 90% prediction interval, using the validation dataset for the 2013 Boston marathon, for the various dropout points along the course. The results for MAE and MSE are directly comparable with those shown in Table 6 for the other procedures considered in this paper.

## Discussion

Although it is difficult to definitively say that any one of our five proposed statistical approaches is better than the others, the nearest neighbor method (KNN) with neighborhoods of size 200 seems competitive with the others based on the measures considered. Alternatively, the “rescaled KNN” method with a slightly smaller value of 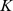 (we have used 100 here) is almost as good based on statistical measures such as MAE or MSE, and is simpler to explain and envision. All five proposed methods clearly perform better than the Constant Pace or Britt methods. Using KNN, the mean absolute error in the prediction of final running time is only about 1.5 minutes, meaning that on average, our predictions should be off by about one and a half minutes compared to the true time we would have seen if the runner had completed the race. In addition, we expect 80% of the runners to receive an estimated finishing time that is within 2 minutes from the true finishing time and 90% of the runners to receive an estimated finishing time that is within 4 minutes from the true one. Within the different subdivisions, female runners are predicted more accurately than male runners, younger runners more accurately than older runners, and faster runners more accurately than slower runners. As expected, the results are much less accurate for runners who had to drop out earlier during the race. Aside from the Constant Pace method, Britt's method appears to perform worse than the others when restricted to runners who stopped at the 35 km or 40 km points. Our final recommendation to the BAA was that they adopt the nearest neighbor prediction “KNN (K = 200)” approach for determining the final results of the 2013 Boston marathon. The file of projected results on our website at http://www.stat.unc.edu/faculty/rs/Bostonwebpage/readme.html includes finishing times under all seven algorithms for each of the 5,524 runners who reached the half marathon point and who were projected by our methods not to have finished before 2:49 pm (the few runners who were projected to have finished by that time we are treating as genuine dropouts, not affected by the bombs). For these runners, we also computed the Boston Qualifier Difference (BQDIF), which measures the difference between the runner's actual time and the official qualifying time for the 2014 Boston marathon based on that runner's age and sex. Runners for whom BQDIF<0 would, by this calculation, have an official qualifying time for 2014. According to our KNN method, there were 158 runners for whom this condition was satisfied. Why did the BAA eventually decide not to use these times for their official results, instead preferring the Constant Pace method? In the end, they made a number of decisions which made the actual predicted results less critical. First among these, they decided to accept *all* the DNF runners for the 2014 race (they had to re-enter the race but were guaranteed acceptance). We estimate that only about 30 runners achieved qualifying times under the BAA projections who would not have done under our proposal (and precisely one runner in the opposite direction), which is too small a number to be worth worrying about. In the end, we can understand the BAA's decision to adopt an approach that is easier to explain and defend, though they acknowledged that our results were informative in helping them make that decision. Nevertheless, our comparisons do show that the alternative methods which we have proposed (in particular, the KNN method in either its original or rescaled form) have considerably better statistical properties than the Constant Pace projections, and we believe they could easily be incorporated into “athlete tracker” apps which are by now widely used in large road races for real-time projections of final results. From a statistical perspective, the analysis provides an example of modern prediction methods based on large datasets, which could well lead to even better methods being developed in the future.
